# A new bioassay for measuring the strength of IL-6/STAT3 signal inhibition by tocilizumab in patients with rheumatoid arthritis

**DOI:** 10.1186/s13075-017-1434-6

**Published:** 2017-10-17

**Authors:** Shuntaro Saito, Katsuya Suzuki, Keiko Yoshimoto, Yuko Kaneko, Yoshihiro Matsumoto, Kunihiro Yamaoka, Tsutomu Takeuchi

**Affiliations:** 1 Division of Rheumatology, Department of Internal Medicine, Keio University School of Medicine, 35 Shinanomachi, Shinjyuku-ku, Tokyo, 160-8582 Japan; 2 Product Research Department, Chugai Pharmaceutical Co., Ltd, 1-1 Nihonbashi-Muromachi 2-Chome, Chuo-ku, Tokyo, 103-8324 Japan

**Keywords:** Rheumatoid arthritis, Tocilizumab, Interleukin-6, Phosphorylated STAT3, CD4+ T cell

## Abstract

**Background:**

Interleukin-6 (IL-6) transduces signals via phosphorylation of STAT3 (pSTAT3). Tocilizumab (TCZ) is an IL-6 receptor blocker, which, when administered intravenously every 4 weeks, efficiently ameliorates rheumatoid arthritis (RA). Since IL-6 signal strength varies among patients with RA, the intensity necessary for appropriate IL-6 signal inhibition by TCZ might vary between individuals. In a previous study, we have examined the clinical utility of increasing (dosing interval shortened to 3 weeks) and decreasing (interval extended to 5 weeks) the dose frequency of TCZ. However, there is currently no established method for accurately measuring the strength of IL-6 signal inhibition by TCZ among individual patients. We therefore sought to develop such an assay.

**Methods:**

Whole blood samples were collected from RA patients with low disease activity (clinical disease activity index (CDAI) ≤ 10) who were treated with TCZ at dosing intervals of 3 weeks (3-week group, *n* = 10), 4 weeks (4-week group, *n* = 10) or 5 weeks (5-week group, *n* = 10), or with methotrexate (control group, *n* = 10). Recombinant human IL-6 (0, 0.1, 1, 10, 100 ng/ml) was exogenously added to whole blood and the proportion of pSTAT3-positive CD4+ T cells (%pSTAT3+/CD4+) was measured by Phosflow cytometric analysis.

**Results:**

The addition of exogenous IL-6 increased the proportion of pSTAT3-positive CD4+ T cells in a dose-dependent manner in each group. Inhibition of IL-6 signaling was strongest in the 3-week dosing group, followed by the 4-week, 5-week and control group. Significant differences in %pSTAT3+/CD4+ cells were observed between dose interval groups when stimulated with 10 ng/ml and 100 ng/ml of IL-6.

**Conclusion:**

Assessment of the proportion of pSTAT3-positive CD4+ T cells under IL-6 stimulation is a highly sensitive and useful method for determining differences in the strength of IL-6 signal inhibition in patients treated with TCZ. It is suggested that different TCZ treatment intervals were necessary to lower disease activity in each group of patients, and these findings also indicate that the IL-6 signaling pathway may differ in each RA patient. Our assay may support strategies for optimizing TCZ treatment in RA patients.

**Electronic supplementary material:**

The online version of this article (doi:10.1186/s13075-017-1434-6) contains supplementary material, which is available to authorized users.

## Background

Interleukin-6 (IL-6) is an inflammatory cytokine with an important role in the pathogenesis of rheumatoid arthritis (RA) [1]. When IL-6 binds to the membrane and soluble forms of the IL-6 receptor (mIL-6R and sIL-6R, respectively), each complex binds to glycoprotein (gp) 130 and induces the phosphorylation of Janus kinase (JAK), which leads to the subsequent phosphorylation of signal transducer and activator of transcription 3 (STAT3) [2]. Phosphorylation of the STAT3 tyrosine residue at position 705 (pY705) is essential for dimerization and nuclear translocation [3].

Tocilizumab (TCZ) is a humanized anti-IL-6 receptor monoclonal antibody that blocks IL-6 signal transduction by binding to mIL-6R and sIL-6R, and efficiently ameliorates RA disease activity at a standard intravenous dose of 8 mg/kg every 4 weeks [4, 5]. Since levels of IL-6 and sIL-6R in serum and synovial fluid vary significantly, IL-6 signal strength varies among patients with RA [6]. Therefore, the intensity necessary for appropriate IL-6 signal inhibition by TCZ might vary between individuals. We previously examined the clinical effect of increasing (dosing interval shortened to 3 weeks) and decreasing (interval extended to 5 weeks) the dose frequency of TCZ to confirm this hypothesis [7]. However, there was no established assay for assessing the strength of IL-6 signal inhibition by TCZ. Such an assay could help to optimize dose frequency and interpret the pharmacokinetics/pharmacodynamics of TCZ. Here, we report the development of an assay for assessing the strength of IL-6 signal inhibition and analysis of the factors associated with IL-6 signaling in RA patients who were treated with TCZ.

## Methods

### Patients

Whole blood samples were collected from a total of 40 patients with RA who met the 1987 American College of Rheumatology (ACR) and/or 2010 ACR/European League Against Rheumatism (EULAR) classification criteria [8, 9] and who had achieved low disease activity (defined by clinical disease activity index (CDAI) [10] ≤ 10) with treatment. Patients were treated with TCZ at dosing intervals of 3 weeks (3-week group, *n* = 10), 4 weeks (4-week group, *n* = 10) or 5 weeks (5-week group, *n* = 10), and those with methotrexate (control group, *n* = 10). Among TCZ-treated patients, TCZ had been administered more than three times in the same interval before blood sample collection.

This study was approved by the ethics committee of our institution (Ethics committee of Keio University School of Medicine, approval number: 20100080 and 20140488, Institutional Review Board of Chugai Pharmaceutical Co., Ltd.). Written informed consent was obtained from all patients. Dosing adjustment of TCZ was performed by the attending physicians with patient consent. The investigation was conducted according to the principles of the Declaration of Helsinki.

### Collection of clinical data

Clinical information was collected from the patients’ medical records, including background characteristics and RA disease activity, methotrexate dose, serum levels of C-reactive protein (CRP), erythrocyte sedimentation rate (ESR), clinical disease activity index (CDAI) [10] and the health assessment questionnaire disability index (HAQ-DI). Clinical and laboratory assessments were performed on the days corresponding to TCZ administration.

### Detection of intracellular pSTAT3

Whole blood samples were collected on the days of TCZ administration, before treatment. Blood samples were stimulated with different concentrations of recombinant human (rh) IL-6 (0, 0.1, 1, 10, 100 ng/ml). The proportion of phosphorylated STAT3 (pSTAT3)-positive cells was measured by phosflow cytometric analysis according to the manufacturer’s protocol (BD Phosflow, Franklin Lakes, NJ, USA). Briefly, whole blood cells from 100 μl of blood sample were stimulated with IL-6 for 15 minutes at 37 °C, and 1 ml of Lyse/Fix Buffer (BD Phosflow) was added for erythrocyte lysis and fixation of the phosphorylated status of STAT3. After fixation, cells were permeabilized with 1 ml of Perm buffer (BD Phosflow), then washed and stained for 60 minutes at room temperature in darkened conditions with the following fluorophore-labeled monoclonal antibodies: anti-CD3-PerCP-Cy5.5 (Biolegend, San Diego, CA, USA), anti-CD4-phycoerythrin (PE)-Cy7 (BD Pharmingen, Franklin Lakes, NJ, USA), anti-pSTAT3 (pY705)-AF488 (BD Phosflow), and anti-mouse immunoglobulin G isotype-matched controls (all from BD Biosciences). Stained cells were washed once with 3 ml of Wash Buffer (BD Phosflow) and analyzed by flow cytometry (MACSQuant Analyzer; Miltenyi Biotec, Bergisch Gladbach, Germany). We also performed analysis of the proportion of pSTAT3-positive CD4+ T cells in healthy individuals (*n* = 2).

### Detection of cell surface expression of mIL-6R and gp130

The surface expression of mIL-6R and gp130 on CD4+ T cells from 50 μl of fresh whole blood was analyzed by flow cytometry on the same day as pSTAT3 detection by phosflow cytometric analysis; this was assessed in a proportion of patients in each group (3-week group, *n* = 5; 4-week group, *n* = 7; 5-week group, *n* = 5; control group, *n* = 8). Expression of mIL-6R and gp130 was assessed by examining the mean fluorescence intensity (MFI) of each molecule. Whole blood cells were stained for 30 minutes at room temperature in darkened conditions with the following fluorophore-labeled monoclonal antibodies: anti-CD3-Vioblue (BD Bioscience), anti-CD4-Viogreen (Miltenyi Biotec), anti-CD126 (mIL-6R)-phycoerythrin (PE, Biolegend), anti-CD130 (gp130)-FITC (Abcam, Cambridge, UK), and anti-mouse immunoglobulin G isotype-matched controls (VioGreen from Miltenyi Biotec, all others from BD Biosciences). Stained cells were washed twice with 2 ml of phosphate-buffered saline and analyzed on a MACSQuant Analyzer (Miltenyi Biotec).

### Measurement of serum IL-6, sIL-6R and TCZ concentrations

The concentrations of IL-6 and sIL-6R in serum were measured by an ultra-sensitive kit (K-15007C-1, MSD, Gaithersburg, MD, USA) and an enzyme-linked immunosorbent assay (ELISA) kit (K151ALC-1, MSD), respectively, according to the manufacturers’ protocol. In order to decrease the interference of rheumatoid factor to ELISA measurement system, we added 200 μg of Immunoglobulin Inhibiting Reagent (6LD1074, Funakoshi Co., Ltd., Tokyo, Japan) to 60 μL of serum sample before the addition of detection antibody. Effective suppression of interference of rheumatoid factor with the addition of this reagent was shown in the report [11]. Serum TCZ levels were measured by a clinical laboratory testing company (SRL Inc., Tokyo, Japan).

### Statistical analysis

Descriptive values are expressed as mean ± standard deviation (SD). Three or more groups were compared using the ruskal–Wallis test and chi-square test. Comparisons between two groups were conducted using the Wilcoxon test and Fisher’s exact test. Correlations were analyzed by Spearman’s correlation coefficient. *P* values <0.05 were regarded as significant. All statistical analyses were performed with JMP software 11.2.0 (SAS Institute, Cary, NC, USA).

## Results

### Patient characteristics

The characteristics of the four groups at the time of blood sample collection are summarized in Table 1. While no significant differences were found in sex, age, RA disease duration, positivity for rheumatoid factor or anti-cyclic citrullinated peptide antibody, the duration of TCZ administration was longer in the 5-week group compared to the other groups. The levels of CRP and ESR were slightly but significantly higher in the control group. There was no statistically significant difference in CDAI between the four groups. The clinical course before blood sample collection in the TCZ-treated groups is summarized in Additional file 1: Figure S1.

**Table 1:** Characteristics of patients who were either administered tocilizumab at different intervals or methotrexate

	3-Week group (*n* = 10)	4-Week group (*n* = 10)	5-Week group (*n* = 10)	Control group (*n* = 10)	*P* value
Sex (female, *n*/total *n*, %)	9/10, 90%	9/10, 90%	8/10, 80%	8/10, 80%	0.85
Age (years)	55.3 (14.8)	65.0 (9.9)	62.0 (10.6)	66.7 (11.0)	0.27
RA duration (months)	101.5 (86.5)	68.5 (74.6)	116.5 (92.5)	81.9 (78.7)	0.35
Tocilizumab duration (months)	27.1 (20.7)	11.9 (9.2)	39.6 (12.1)	-	<0.01*
RF positive (*n*, %)	7/10, 70%	9/10, 90%	9/10, 90%	5/10, 50%	0.12
ACPA positive (*n*, %)	5/8, 63%	7/10, 70%	8/10, 80%	6/10, 60%	0.77
MTX use (*n*, %)	4/10, 40%	6/10, 60%	4/10, 40%	10/10, 100%	0.03*
MTX dose (mg/week)	7.5 (4.1)	8.3 (2.0)	4.4 (0.9)	9.0 (3.6)	0.07
CRP (mg/dl)	0.01 (0.02)	0.01 (0.02)	0.03 (0.03)	0.13 (0.10)	<0.01*
ESR (mm/h)	6.0 (4.7)	6.6 (5.6)	12.3 (14.4)	22.2 (12.8)	<0.01*
CDAI	5.2 (1.3-8.1)	1.7 (0.1-7.9)	1.7 (0.1-5.8)	3.6 (0.2-6.3)	0.07
HAQ-DI	1.1 (0.8)	0.5 (0.6)	0.3 (0.5)	0.5 (0.5)	0.06

Values are expressed as mean ± standard deviation (SD) unless stated otherwise

*RA* rheumatoid arthritis, *RF* rheumatoid factor, *ACPA* anti-cyclic citrullinated peptide antibody, *MTX* methotrexate, *CRP* C-reactive protein, *ESR* erythrocyte sedimentation rate, *CDAI* clinical disease activity index, *HAQ-DI* health assessment questionnaire disability index

*P < 0.05

### Proportion of pSTAT3-positive CD4+ T cells

**Fig. 1:**
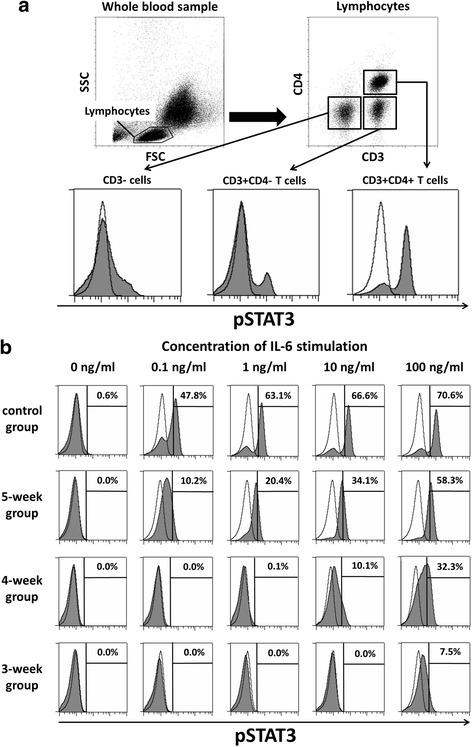
Assessment of the strength of interleukin-6/phosphorylated STAT3 (IL-6/pSTAT3) signal inhibition. **a** Representative intracellular staining pattern of pSTAT3 in CD3+ cells, CD3 + CD4− T cells and CD3 + CD4+ T cells by flow cytometry (IL-6 stimulation = 100 ng/ml). **b** Measurement of the proportion of pSTAT3-positive CD4+ T cells with exogenous IL-6 stimulation (0, 0.1, 1, 10, 100 ng/ml) in each group of patients administered either tocilizumab (TCZ) at different intervals or methotrexate (MTX). Open histograms represent isotype control antibody staining and filled plots represent anti-pSTAT3 antibody staining

**Fig. 2:**
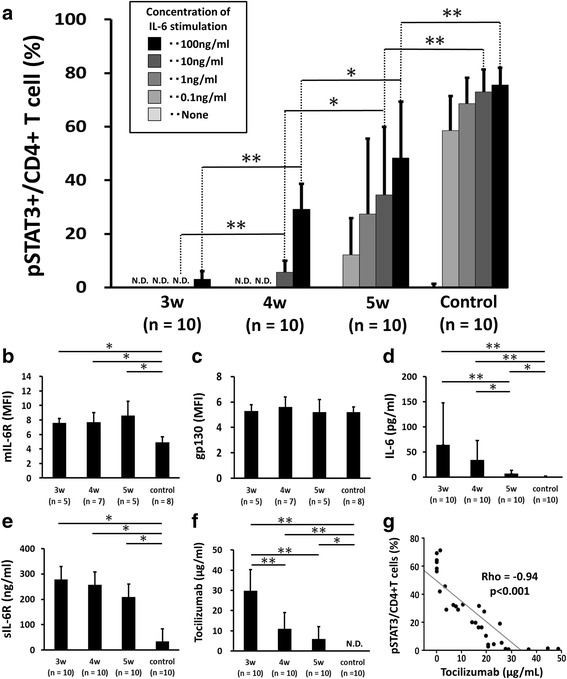
Difference in phosphorylated STAT3 (pSTAT3)+/CD4+ T cells (%) and other factors associated with interleukin-6 (IL-6) signaling in each group of patients administered tocilizumab (TCZ) at different intervals and in the control group. Comparison of pSTAT3+/CD4+ T cells (%) under IL-6 stimulation (0, 0.1, 1, 10, 100 ng/ml) (**a**), mean fluorescence intensity (MFI) of the membrane form of IL-6 receptor (mIL-6R) in CD4+ T cells (**b**), MFI of gp130 in CD4+ T cells (**c**), serum IL-6 concentration (**d**), serum soluble form of IL-6 receptor (sIL-6R) concentration (**e**) and serum TCZ concentration (**f**) in each treatment group. **g** Correlation between serum TCZ concentration and pSTAT3+/CD4+ T cells (%; IL-6 stimulation = 100 ng/ml) in the TCZ-treated group (n = 30). Wilcoxon test was used for comparing the groups. Correlation was analyzed by Spearman’s correlation coefficient. w = week

A representative figure showing the detection of pSTAT3-positive cells in whole blood from patients in each group is shown in Fig. 1a. Since CD4+ T cells were the most sensitive to IL-6 stimulation compared with CD3 + CD4− T cells and CD3− cells (Fig. 1a), we focused on the proportion of pSTAT3-positive CD4+ T cells in the assay. The proportion of pSTAT3-positive CD4+ T cells (%pSTAT3+/CD4+) increased with exogenous rhIL-6 stimulation in a dose-dependent manner in each treatment group (Fig. 2a). Although all patients had low disease activity, inhibition of IL-6 signaling was strongest in the 3-week group, followed by the 4-week, 5-week and control group for every IL-6 concentration (Fig. 2a).

In the 3-week group, %pSTAT3+/CD4+ was 0 ± 0.0%, suggesting complete inhibition of IL-6 signaling, with IL-6 concentrations of 0 to 10 ng/ml. This increased to 3.1 ± 3.0% with 100 ng/ml of IL-6, indicating low signal transduction. Similarly, in the 4-week group, complete inhibition of IL-6 signaling was observed at IL-6 concentrations of 0 to 1 ng/ml, while low levels of IL-6 signal transduction (%pSTAT3+/CD4+ = 5.6 ± 4.4%) were observed with 10 ng/ml of IL-6. In the 5-week group, %pSTAT3+/CD4+ was 12.2 ± 13.7% with 0.1 ng/ml of IL-6, and reached 27.3 ± 28.1% with 1 ng/ml of IL-6, although there was wide variation in this group. In the control group, very low levels of IL-6 signal transduction (%pSTAT3+/CD4+ = 0.8 ± 1.3%) were observed without exogenous IL-6 stimulation (0 ng/ml), and %pSTAT3+/CD4+ reached 58.6 ± 12.8% with 0.1 ng/ml of IL-6. Notably, there were significant differences in %pSTAT3+/CD4+ between combinations of any two groups at 10 ng/ml and 100 ng/ml concentrations of IL-6 stimulation (Fig. 2a). In addition, there was no significant difference in the proportion of pSTAT3-positive CD4+ T cells between healthy individuals and RA control patients treated without anti-IL6-R. The proportion of pSTAT3-positive CD4+ T cells in healthy individuals were included in the range of mean ± 2 SD of the RA control group, and was significantly higher than that in the 5-week group, in all concentrations of IL-6 stimulation (data not shown).

### Other factors associated with IL-6 signaling

#### Surface mIL-6R and gp130 expression in CD4+ T cells

Although the MFI of mIL-6R on CD4+ T cells from the TCZ-treated groups was higher than that from the control group (*p* < 0.05 for all comparisons), there was no significant difference between the 3-week, 4-week or 5-week groups (Fig. 2b). The MFI levels of gp130 on CD4+ T cells were comparable among all groups (Fig. 2c).

#### Serum IL-6 and sIL-6R levels

The concentration of serum IL-6 was significantly higher in the 3-week and 4-week groups compared to the 5-week and control groups. Although IL-6 concentration in the 5-week group was significantly higher than that in the control group, it was not significantly different between the 3-week and 4-week groups (*p* = 0.21, Fig. 2d). The concentration of sIL-6R was higher in the TCZ-treated group than in the control group (*p* < 0.05 for all comparisons), but there was no significant difference between the 3-week, 4-week, or 5-week groups (Fig. 2e).

#### Serum TCZ level

The concentration of serum TCZ was significantly higher in the 3-week group compared to all other groups (*p* < 0.05 for all comparisons). While TCZ concentration in the 4-week and 5-week groups was significantly higher than that in the control group (*p* < 0.05 for all comparisons), there was no significant difference between the 4-week and 5-week groups (*p* = 0.09; Fig. 2f). There was strong and significant inverse correlation (Rho = − 0.94, *p* < 0.001) between serum TCZ and %pSTAT3+/CD4+ (IL-6 stimulation = 100 ng/ml), with a notable difference detected at low TCZ concentrations (Fig. 2g).

## Discussion

We demonstrated that assessing the proportion of pSTAT3-positive CD4+ T cells under IL-6 stimulation is a highly sensitive and useful method for determining the strength of IL-6 signal inhibition between groups of patients who were administered TCZ at different intervals and the MTX-treated control group. There was no significant difference in the serum IL-6 concentration between the 3-week and 4-week groups. Serum IL-6 concentrations had different dynamics compared with %pSTAT3+/CD4+ T cells, suggesting that the proportion of pSTAT3-positive CD4+ T cells was not directly affected by serum IL-6. Despite strong inverse correlation between serum TCZ concentration and %pSTAT3+/CD4+ T cells, there was no significant difference in serum TCZ concentration between the 4-week and 5-week groups. This suggests that the proportion of pSTAT3-positive CD4+ T cells may not be directly affected by free serum-TCZ. Instead, it may be correlated with the binding rate of TCZ to the IL-6 receptor; however, we could not measure the binding rate in this study. This novel pSTAT3-positive CD4+ T cell assay was more sensitive for assessing the strength of IL-6 signal inhibition compared to measuring serum TCZ concentrations. Although sIL-6 concentration and mIL-6R expression on CD4+ T cells were higher in the TCZ-treated group than in the control group, there was no significant difference between the TCZ-treated groups. We hypothesized that the mIL-6R expression on CD4+ T cells may have been induced by a positive feedback mechanism in response to IL-6 signal inhibition, and that these values plateaued in each group. In addition, increased sIL-6R concentration may have been affected by reduced clearance rate by TCZ binding. Interestingly, gp130 expression in CD4+ T cells was equivalent in all TCZ-treated groups and the control group, suggesting that gp130 expression on CD4+ T cells is not affected by inhibition of IL-6 signal.

Our study demonstrated that the strength of IL-6/pSTAT3 inhibition differed depending on the interval of TCZ administration, which all resulted in low disease activity in each group of patients with RA. Therefore, it is suggested that different TCZ treatment intervals were necessary to lower disease activity in each group of patients. These findings also indicate that the IL-6 signaling pathway may differ in each patient with RA, further supporting the applicability of an assay for assessing pSTAT3-positive CD4+ T cells under IL-6 stimulation as a tool for adjusting the TCZ interval in patients with RA.

This is the first study to assess the dynamic status of molecules associated with IL-6 signaling with respect to the dose frequency of TCZ. Since the novel assay system utilized in this study uses whole blood that contains TCZ, detection of the proportion of pSTAT3-positive CD4+ T cells reflects the strength of IL-6 signal inhibition by TCZ in vivo, and it is a unique system for assessing patients with RA under TCZ treatment. In addition, it is possible that the assay for pSTAT3-positive CD4+ T cells may reflect the strength of IL-6 signal inhibition by TCZ in the articular environment; however, since there are few studies on the articular concentration of TCZ, further studies are required to confirm this. The advantage of this assay is its high sensitivity for detecting differences in the strength of IL-6 signal inhibition, which could be applicable to other receptor blockade therapies.

We should note some limitations of our study. First, we could not assess the binding rate of TCZ to mIL-6R and sIL-6R; therefore, we could not determine the most influential factor for pSTAT3-positive CD4+ T cells. Second, although the detection rate of anti-TCZ antibodies has previously been reported to be infrequent [12], future studies should examine this further. Third, the ELISA method we utilized could not discriminate free IL-6 from IL-6 bound to its receptor, sIL-6R, nor free sIL-6R from sIL-6R bound to IL-6 and/or anti-IL-6R antibody: the data on IL-6 and sIL-6R represented the total amounts of these factors.

Since the variation of %pSTAT3/CD4+ T cells within each group of TCZ-treated patients was relatively large, the pharmacokinetics/pharmacodynamics of TCZ might differ among individuals. Therefore, measurement of the proportion of pSTAT3-positive CD4+ T cells under IL-6 stimulation might support strategies to optimize treatment in patients with RA treated with TCZ. Further studies are required to establish the clinical usefulness of assessing the strength of IL-6 signal inhibition with this assay in patients who require TCZ dose frequency adjustments.

## Conclusions

We developed a highly sensitive bioassay for measuring the strength of IL-6/STAT3 signal inhibition by tocilizumab in patients with RA. This bioassay system may support strategies for optimizing TCZ treatment in patients with RA.

## Additional file

Additional file 1: Figure S1. Transition in CDAI for each group of patients administered TCZ at different intervals. Transition in CDAI for the 3-week group (**A**), 4-week group (**B**) and 5-week group (**C**). *TCZ* tocilizumab, *CDAI* clinical disease activity index. (TIF 1404 kb)

## Abbreviations

CDAI: Clinical disease activity index
CRP: C-reactive protein
ELISA: Enzyme-linked immunosorbent assay
ESR: Erythrocyte sedimentation rate
gp130: Glycoprotein 130
HAQ-DI: Health assessment questionnaire disability index
IL-6: Interleukin-6
JAK: Janus kinase
MFI: Mean fluorescence intensity
mIL-6R: Membrane IL-6 receptor
pSTAT3: Phosphorylated signal transducer and activator of transcription 3
RA: Rheumatoid arthritis
sIL-6R: Soluble IL-6 receptor
TCZ: Tocilizumab
