# Supplementary figures and images for: A new bioassay for measuring the strength of IL-6/STAT3 signal inhibition by tocilizumab in patients with rheumatoid arthritis
Source: Arthritis Res Ther. 2017 Oct 17;19:231. doi: 10.1186/s13075-017-1434-6 (PMC5645925; doi:10.1186/s13075-017-1434-6)

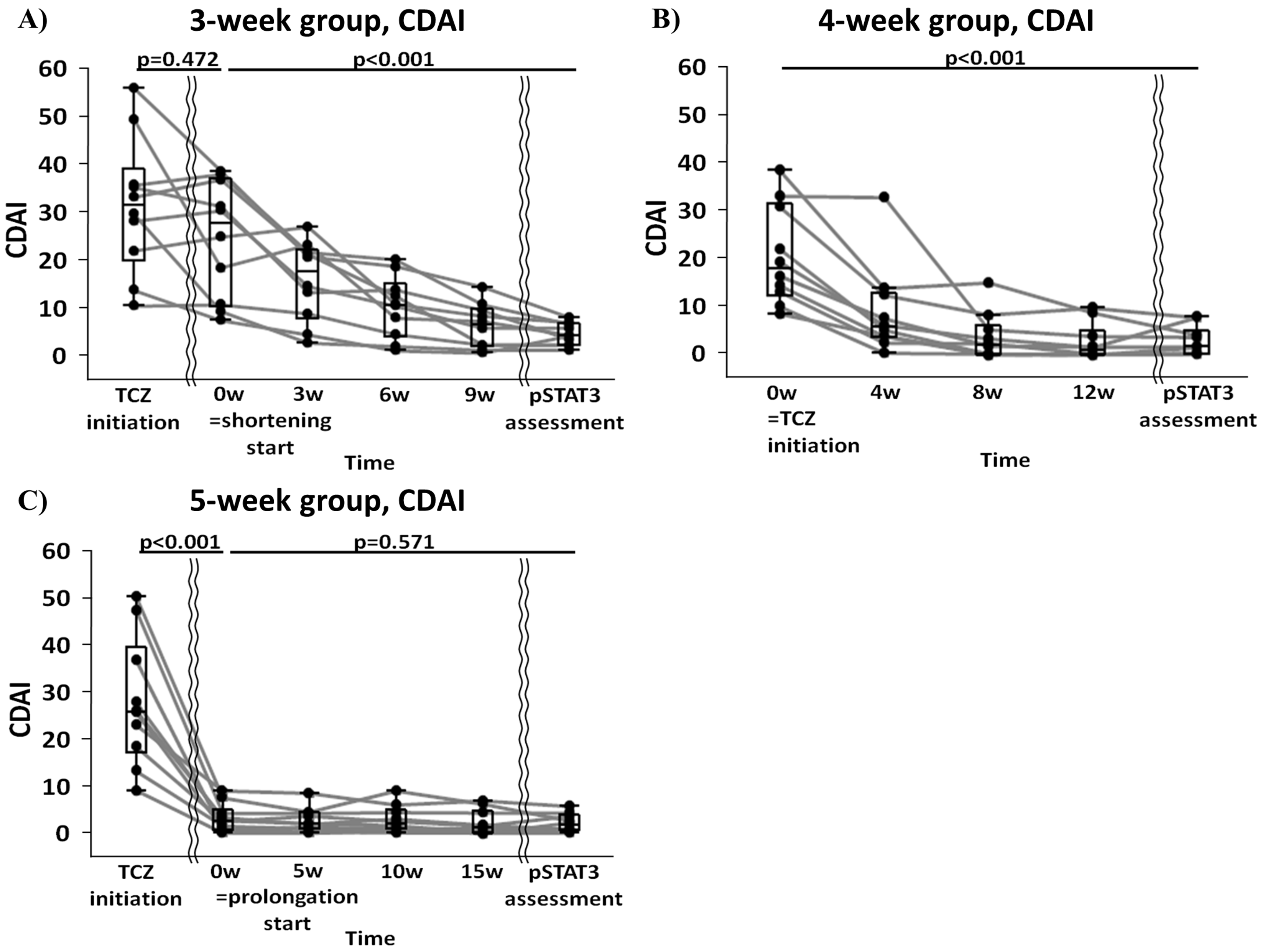

Supplement: Additional file 1: Figure S1. — Transition in CDAI for each group of patients administered TCZ at different intervals. Transition in CDAI for the 3-week group (A), 4-week group (B) and 5-week group (C). TCZ tocilizumab, CDAI clinical disease activity index. (TIF 1404 kb) [file 13075_2017_1434_MOESM1_ESM.tif]
